# A Genome-Wide Analysis of the Pentatricopeptide Repeat Protein Gene Family in Two Kiwifruit Species with an Emphasis on the Role of RNA Editing in Pathogen Stress

**DOI:** 10.3390/ijms241813700

**Published:** 2023-09-05

**Authors:** Aidi Zhang, Yuhong Xiong, Fang Liu, Xiujun Zhang

**Affiliations:** 1 Key Laboratory of Plant Germplasm Enhancement and Specialty Agriculture, Wuhan Botanical Garden, Chinese Academy of Sciences, Wuhan 430074, China; zhangaidi@wbgcas.cn (A.Z.); xiongyuhong20@mails.ucas.ac.cn (Y.X.); liufang@wbgcas.cn (F.L.); 2 Center of Economic Botany, Core Botanical Gardens, Chinese Academy of Sciences, Wuhan 430074, China; 3 University of Chinese Academy of Sciences, Beijing 100049, China

**Keywords:** pentatricopeptide repeat protein, pathogens infection, RNA editing, kiwifruit

## Abstract

Kiwifruit is a perennial fruit tree with high nutritional and economic value; however, various pathogen stresses have resulted in reductions in its yield and quality. Pentatricopeptide repeat proteins (PPRs), characterized by tandem repetitions of 35 amino acid motifs, play roles in RNA editing, mRNA stability, and splicing. They may also regulate plant development and growth. Nevertheless, the roles of PPRs in plant development and disease resistance remain unclear. In this study, we focused on the roles of PPRs in the fruit development and pathogen stress of kiwifruit and conducted a series of analyses of the PPR gene family in two representative kiwifruit species (*Actinidia chinensis* (*Ach*) and *Actinidia eriantha* (*Ace*)) with markedly different degrees of disease resistance. A total of 497 and 499 PPRs were identified in *Ach* and *Ace*, respectively. All the kiwifruit PPRs could be phylogenetically divided into four subfamilies. There were about 40.68% PPRs predicted to be localized to mitochondria or chloroplasts. A synteny analysis showed that the expansion of the kiwifruit PPRs mainly originated from segmental duplication. Based on RNA-seq data from the fruit over 12 periods of development and maturity, a weighted correlation network analysis suggested that two PPRs, Actinidia20495.t1 and Actinidia15159.t1, may be involved in fruit development and maturation. In addition, we observed different responses with respect to the expression of PPRs and RNA editing between resistant and susceptible kiwifruits following infection with pathogenic bacteria, indicating the regulatory role of PPRs in the stress response via the modulation of RNA editing. The differentially expressed upstream transcription factors of the PPRs were further identified; they may regulate resistance adaption by modulating the expression of the PPRs. Collectively, these results suggest that PPRs play roles in the development and disease resistance of kiwifruit and provide candidate genes for further clarifying the resistance mechanisms in kiwifruits.

## 1. Introduction

Kiwifruit (*Actinidia chinensis*, *Ach*), also called the Chinese gooseberry, is a type of popular horticultural fruit rich in vitamin C, dietary fiber, minerals, and other nutrients beneficial for health. Due to its enormous nutritional and economic value, the kiwifruit industry has a promising future [1]. However, various diseases have adversely affected its quality and yield. Bacterial canker, which is caused by the bacterial pathogen *Pseudomonas syringae* pv. *actinidiae* (Psa), has become one of the severe threats in the cultivation and production of kiwifruit [2]. Psa can degrade lignins and phenols, and infected trees exhibit typical symptoms comprising necrotic spots, blossom necrosis, and twig wilting, which severely threaten the production of kiwifruit. The process of infection of bacterial canker in kiwifruit causes rapid deterioration without any effective rescue. Bacterial canker was first reported on *Actinidiae chinensis* var. *deliciosa* in Shizuoka, Japan, in 1984, and today, it has been reported worldwide, including in China, Chile, and European countries [3,4,5]. Most current kiwifruit cultivars are mainly derived from *Actinidia chinensis* (*Ach*), such as ‘HongYang’, ‘Hort 16A’, ‘Gold3’, and ‘Hayward’, which are generally sensitive to Psa. However, there are still certain kiwifruit species or cultivars that show resistance to Psa, such as *Actinidia eriantha (Ace)* cv. ‘HuaTe’. The pathogen stress represented by bacterial canker has caused severe economic losses to the kiwifruit production industry. Therefore, it is of great significance to study the disease resistance mechanism and breed a resistant germplasm in kiwifruit.

RNA editing is a typical post-transcriptional modification in which a pre-mRNA sequence undergoes a nucleotide insertion/deletion or conversion [2]. In plants, RNA editing only occurs in mRNA transcribed by the genomes from plastids and mitochondria [6,7,8]. There are usually 400–500 and 30–40 C-to-U (cytosine-to-uracil) conversions in the transcripts of mitochondria and plastids, respectively [9]. RNA editing is a crucial process in maintaining the normal functions of encoded proteins at the mRNA level, and it plays important roles in various plant developmental processes, including organelle biogenesis, reproductive development, and adaptation to environmental changes [10,11]. Different RNA editing responses to oxidative stress were detected between the wild-abortive male-sterile line and its maintainer line [12]. The dynamic response of plant RNA editing to environmental factors, such as salt and heat stresses, was also detected in several recent studies [13,14]. Additionally, recent research has demonstrated that RNA editing is responsive to pathogen stress and can affect disease resistance [15,16,17].

In plants, numerous studies have proven that a number of factors mediate RNA editing, such as pentatricopeptide repeat proteins (PPRs), organelle RNA recognition motif-containing proteins (ORRMs), protoporphyrinogen IX oxidases (PPOs), and multiple organellar RNA editing factors (MORFs), which comprise an RNA editing complex and interact with each other to mediate RNA editing [10,18,19]. Among these factors, PPRs function as RNA-binding proteins that directly interact with the *cis*-elements of the target mRNA and determine the specificity of RNA editing. Typically, one PPR protein specifically recognizes one or several RNA editing sites [20]. PPRs contain a series of tandem array repeats characterized by a 35-amino-acid (aa) helix-turn-helix motif. The number of tandem repeats generally ranges from 2 to 27 copies in the recently characterized PPR gene family. Based on the variation in the repeat composition, PPRs can be classified into P-type and PLS-type subfamilies. The ancestral P-type subfamily only contains the canonical PPR motifs adjacent to each other without other motifs, whereas the PLS subfamily consists of PPR motifs interspaced by two PPR-like motifs, the shorter PPR-like motif (PPR-like S, 31–34 aa) and the longer PPR-like motif (PPR-like L, 35–37 aa). However, PPR motifs are mainly classified depending on the presence of conserved motifs rather than their sheer length, leading to the distinction of multiple PPR subgroups (e.g., P1 and P2; L1, L2, and LL; and S1, S2, and SS). Sequence alignments of different PPR and PPR-like motifs reveal the conserved sequences [21]. Based on the C-terminal composition, PLS-type PPRs can be divided into three smaller subclasses, including the PPR-E, PPR-E+, and PPR-DYW subtypes. The PPR proteins involved in RNA editing belong to the PLS-type subfamily. In PPR-DYW proteins, the DYW domain provides cytidine deaminase activity, whereas PPR-E+ proteins recruit an atypical PPR-DYW protein to function in RNA editing [22]. Approximately 200 PLS-type PPRs were proven to be involved in RNA editing in Arabidopsis [20].

PPRs are widely distributed across various plant lineages, comprising more than 400 family members. They have been identified in many different plants, with 450, 477, and 486 members of PPRs predicted in the genomes of Arabidopsis, rice, and foxtail, respectively [23]. Generally, PPRs function as organelle-specific RNA-binding proteins and play roles in multiple organelle functions and biological processes, such as the regulation of gene expression, RNA exon splicing, editing, and stability, and the translation of RNAs [24,25,26]. In general, the P-type PPRs mainly function in most aspects of organelle gene expression, RNA stabilization, and splicing, whereas the PLS-type PPRs mainly function in RNA editing [27]. Deficiencies in the functions of PPRs often result in organelle dysfunction, defects in growth and embryo development, or abnormal stress sensitivity [22,28,29,30,31,32]. *OsPPR11* has been proven to function in chloroplast development by affecting group II intron splicing in rice [33]. PPRs have also been reported to be involved in responses to various biotic and abiotic stresses. For instance, the PPR protein SOAR1 has been proven to regulate salt tolerance in rice [31]. With the development of synthetic RNA biology, PPRs can be harnessed for the manipulation of intron splicing or the stabilization of organellar RNA molecules, and these engineered PPRs will potentially be applicable to improving plants’ fitness in response to developmental and environmental cues.

However, the functions of PPRs in kiwifruit, especially their roles in disease resistance, remain unclear. Furthermore, the underlying roles of RNA editing are not fully understood. Accordingly, in this study, we conducted a comprehensive analysis to evaluate the evolution, expansion, distribution, and expression patterns of PPRs in kiwifruit, particularly focusing on the responses of expression and RNA editing profiles to pathogen infection. Based on the results, we observed that PPRs and RNA editing exhibited a noticeable response to infection, with differing responses detected in two kiwifruit varieties with different resistance levels. Additionally, we explored candidate upstream transcription factors that may regulate responses by modulating PPR gene expression. Our findings provide insights into the biological functions of PPRs in response to pathogen stress in kiwifruit and will contribute to a better understanding of the roles of RNA editing in plant immunity.

## 2. Results

### 2.1. Identification and Synteny Analysis of PPR Members in Two Kiwifruit Species

**Figure 1:**
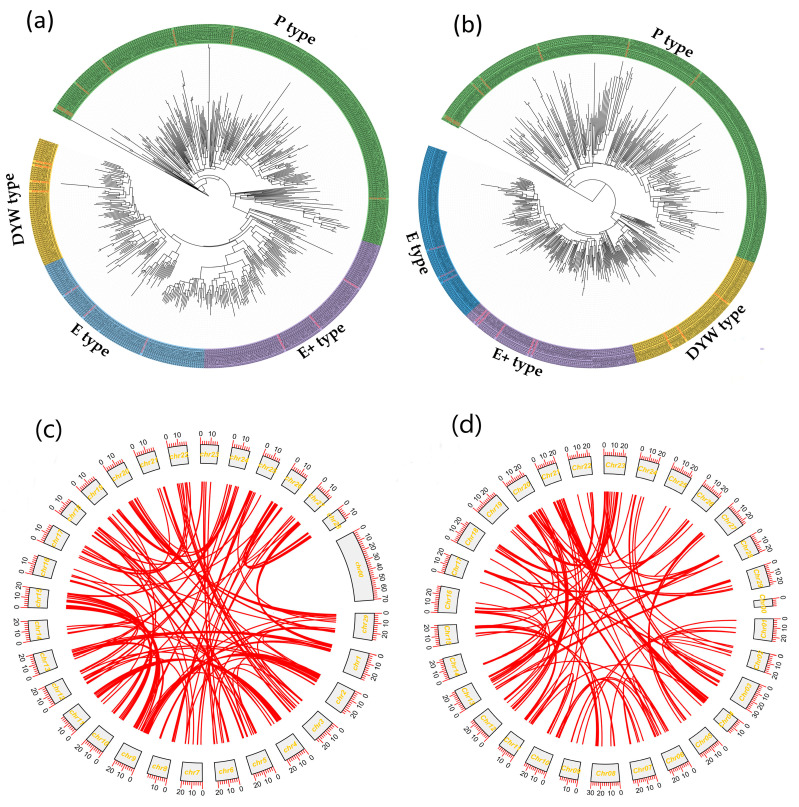
The phylogenetic relationships and self-collinearity of PPRs in *Actinidia chinensis* (*Ach*) and *Actinidia eriantha* (*Ace*). A total of 497 and 499 PPRs in *Ach* and *Ace* were used for constructing a phylogenetic tree via the maximum likelihood (ML) method [38]. All the PPRs were classified into four types, and different groups are shaded in different colors: the green, blue, purple, and yellow areas represent the P, E, E+, and DYW types of PPRs, respectively. The names of representative PPRs in Arabidopsis are highlighted in red fonts. (**a**) The phylogenetic relationships of PPRs in *Ach.* (**b**) The phylogenetic relationships of PPRs in *Ace.* (**c**) The interchromosomal relationships of PPRs in *Ach*. (**d**) The interchromosomal relationships of PPRs in *Ace*. The red lines highlight the syntenic PPR pairs.

We searched the *Ach* genome using 450 known Arabidopsis PPRs as queries. Through HMM [34] and BLASTP searches, we identified 644 and 497 putative *Ach* proteins, respectively. We retained the 497 overlapping PPRs that were identified in both searches as PPRs in *Ach*. Similarly, we identified 499 putative PPRs in *Ace*. Detailed information about the results of the BLAST searches is presented in Tables S1 and S2. Based on their phylogenetic relationships from protein sequences (Figure 1), all the PPRs were classified into four clades, including P- and PLS-types (E-type, E+-type, and DYW-type), with 251, 91, 95, and 60 in *Ach*, and 247, 74, 102, and 76 in *Ace*, respectively. In addition, the types of the identified PPRs were further verified through their phylogenetic relationships with known representative PPRs in Arabidopsis from different clades. Among the *Ach* PPRs, P-type PPRs were the most abundant, while DYW-type PPRs were the least common. Conversely, in *Ace*, P-type PPRs were the most abundant, and E-class PPRs were the least common (Table 1). We used a combination of TargetP2.0 [35] and Predotar4.0 [36] to predict the subcellular localization of PPRs. The results showed that 40.64% were localized in mitochondria or chloroplasts in *Ach*, with 28.37% of the PPRs located in mitochondria, and 12.27% in chloroplasts. For *Ace*, 43.88% were located in mitochondria or chloroplasts, with 32.06% of the PPRs in mitochondria, and 11.82% in chloroplasts. The number of amino acids ranged from 91 to 1871 in *Ach*, and from 111 to 1738 in *Ace* (Tables S3 and S4). The chromosomal localization results revealed that kiwifruit PPRs were widely distributed across nearly all of the chromosomes (Figure S1).

Synteny analysis was further performed to determine the duplication events and possible collinear blocks within the kiwifruit genome or between genomes. The collinearity analysis of the PPRs between the genomes of *Ach* and Arabidopsis showed that 180 *Ach* PPRs exhibited syntenic relationships with 179 PPRs of Arabidopsis, while 155 *Ace* PPRs exhibited syntenic relationships with 154 PPRs in Arabidopsis, involving all four types of PPRs, indicating the evolutionary conservation of PPRs (Table S5). Synteny analysis within the genomes showed that kiwifruit PPRs exhibited high homologous conservation, as depicted in Figure 1. The origins of duplicated genes in the kiwifruit genome were further classified into five categories, including whole-genome/segmental duplications where matching genes were located in syntenic blocks, tandem duplications (characterized by continuous repeats), proximal duplications (genes in nearby chromosomal regions but not adjacent), or dispersed duplications (occurring through other mechanisms). The results indicated that the origins of duplicated PPRs in *Ach* were divided into 11 singletons, 24 dispersed, 8 proximal, 9 tandem, and 445 segmental duplications (Table S3). The PPRs in *Ace* exhibited a similar distribution. The above results suggest that the expansion of kiwifruit PPRs mainly resulted from segmental duplication, often accompanied by whole-genome duplication.

The Ka/Ks values were further calculated for the paralogous PPRs to assess the selection pressure during evolution. Approximately 60 pairs of paralogous PPRs were obtained from MCScanX [37] for each kiwifruit species, and all the Ka/Ks values for these gene paralogs were less than one, indicating that these genes evolved slowly after duplication under purifying selection (Table S6). Additionally, we identified the orthologous PPRs between *Ach* and *Ace*, obtaining a total of 306 pairs from MCScanX. Only two pairs exhibited Ka/Ks values greater than one, including the pairs Actinidia00869-DTZ79_13g12210 and Actinidia08538-DTZ79_28g04010. The former belongs to P-type PPRs, and the latter belongs to E+-type PPRs (Table S7). This observation suggests that the PPRs evolved slowly after the speciation of *Ach* and *Ace*.

### 2.2. PPRs Play Roles in Fruit Development and Ripening

**Figure 2:**
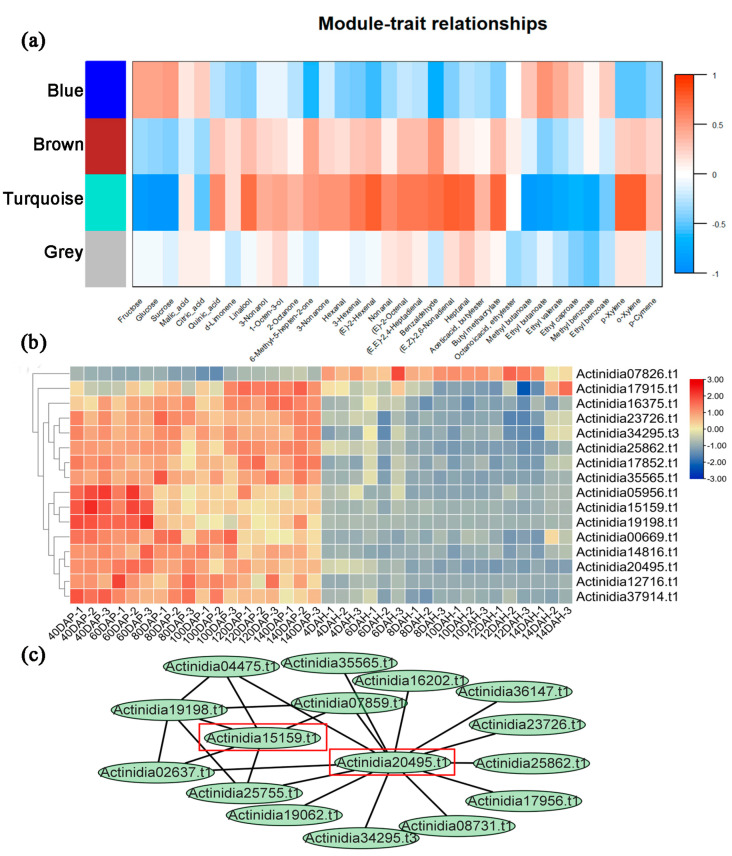
Expression analysis of PPRs during fruit development and ripening in *Actinidia chinensis* (*Ach*). (**a**) Module-feature clustering based on the expression of PPRs during fruit development and maturation of *Ach.* The heatmap color gradient indicates the level of correlation density, with red indicating a positive correlation and blue indicating a negative correlation. (**b**) Gene expression heatmap of PPRs during fruit development and maturation of *Ach*. The gene expression levels were measured using FPKM (fragments per kilobase of transcript per million mapped reads), and subjected to log2 transformation and row-scale normalization. The *x*-axis represents six developmental periods (40, 60, 80, 100, 120, and 140 days after pollination (DAP)) and six maturity periods (4, 6, 8, 10, 12, and 14 days after harvest of ripe fruit (DAH)). (**c**) Co-expression network of PPRs during fruit development and maturation of *Ach*, with two hub PPRs highlighted with red rectangles.

Based on the RNA-seq data [39] obtained during fruit development and maturation in *Ach*, we analyzed the gene expression profiles of PPRs in kiwifruit across different time periods. After preliminary screening, a total of 260 PPRs exhibited expression during the fruit development and maturation stages in *Ach*. Most PPRs were highly expressed during the development stage and showed lower expression levels after harvest, with only a small subset of PPRs displaying higher expression at the maturity stage. Sixteen PPRs exhibited differential expression at various time points (Figure 2). Regarding the types of PPRs, the expression of PLS-type PPRs was higher during the development stage, while the expression of P-type PPRs was higher during the maturity stage. Module-feature clustering was conducted based on the expression patterns of PPRs during kiwifruit development and maturation. According to the results of weighted gene co-expression network analysis (WGCNA), the PPRs were grouped into four modules (turquoise, blue, brown, and grey), consisting of 188, 7, 5, and 60 PPRs, respectively (Table S8). The results indicated that gene expression in the turquoise module was primarily associated with traits such as glucose, quinic acid, and linalool. The expression of PPRs in this module showed a negative correlation with the levels of sugars (such as glucose) and esters (such as ethyl butyrate) while displaying a positive correlation with the levels of alcohols (such as linalool) and acids (such as quinic acid) (Figure 2). In other words, during the fruit development and maturation stages, PPRs in the turquoise module had a negative regulatory effect on the synthesis of glucose, ethyl butyrate, and other esters and sugars, but had a positive regulatory effect on the synthesis of quinic acid, linalool, etc. Regarding the relationships between different modules, the turquoise module exhibited a positive correlation with genes in the brown module and a negative correlation with genes in the blue module, while genes in the gray module did not show significant associations with those in any other modules.

Furthermore, we utilized the turquoise module to identify the hub PPRs with higher degrees within the module. These hub PPRs can better represent the overall expression pattern of genes in this module and exhibit stronger correlations with specific characteristics (Figure 2). Hub genes were selected based on a module membership closeness greater than 0.8. Consequently, we identified 72, 4, 7, and 11 hub genes related to glucose, quinic acid, linalool, and ethyl butanoate, respectively. Some of these hub PPRs were associated with multiple characteristic metabolites. For instance, *Actinidia20495.t1* served as a hub gene in regulating the synthesis of glucose, linalool, and ethyl butanoate. *Actinidia15159.t1* was identified as a hub gene involved in regulating the synthesis of glucose, quinic acid, and linalool. *Actinidia14816.t1* and three other PPRs were hub genes for the synthesis of glucose and linalool. *Actinidia07826.t1* and other PPRs were hub genes associated with glucose and ethyl butanoate. *Actinidia19198.t1* served as a hub gene in regulating the synthesis of quinic acid and linalool, while *Actinidia05956.t1* was a hub gene for the synthesis of glucose and quinic acid. The PPRs that regulated glucose synthesis were closely related to each other. Module membership-gene characteristics associated with glucose, quinic acid, linalool, and ethyl butyrate in kiwifruit are shown in Figure S2. In conclusion, we hypothesize that *Actinidia20495.t1* and *Actinidia15159.t1* are involved in the fruit development and maturation of kiwifruit. They may play roles in the synthesis of sugar and acid compounds, as well as other changes in the content of alcohols (such as linalool) and esters (like ethyl butyrate) during kiwifruit development and ripening. The orthologs of *Actinidia20495.t1* and *Actinidia15159.t1* are *PCMP-E48* and *PCMP-E88* in Arabidopsis, both of which belong to the plant combinatorial and modular protein (PCMP) subfamily, specific to land plants. They mainly function in RNA binding and modification. However, their underlying molecular functions in fruit development remain to be explored.

### 2.3. Different PPR Expression and RNA Editing in Response to Psa Infection in Two Kiwifruit Species

**Figure 3:**
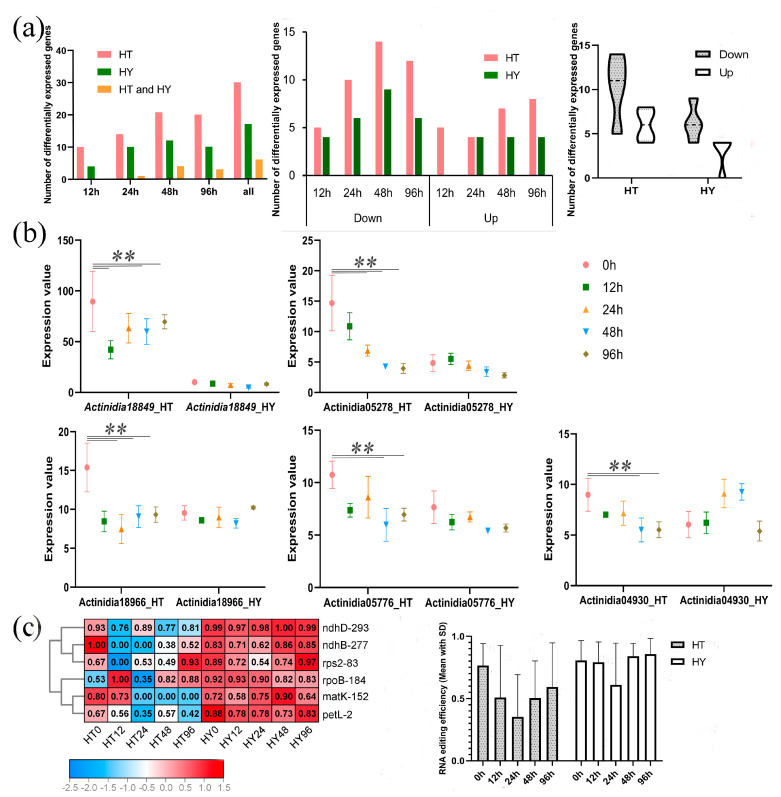
Different responses of PPRs expression and RNA editing to Psa infection in *Actinidia chinensis* (*Ach*) and *Actinidia eriantha* (*Ace*). (**a**) The number of differentially expressed PPRs and up–down-regulated PPRs after Psa infection in two kiwifruit species. (**b**) Gene expression of PPRs between resistant (*Ace*) and susceptible (*Ach*) kiwifruits after *Psa* infection. The *x*-axis denotes hours after Psa infection (0, 12, 24, 48, and 96 hai), and the *y*-axis denotes the PPRs. The asterisk denotes significant differences: ** *p*-value < 0.01. (**c**) RNA editing profiles in two kiwifruit species. The *x*-axis represents hours after Psa infection, and the *y*-axis represents RNA editing frequency. ‘HongYang’ (HY) and ‘HuaTe’ (HT) represent susceptible and resistant kiwifruits, respectively.

Based on RNA-seq data [40] from resistant (‘HuaTe’, *Ace*) and susceptible (‘HongYang’, *Ach*) kiwifruits during early infection of *Pseudomonas syringae pv. Actinidiae* (Psa), we analyzed the expression of PPRs between these two different kiwifruit species (Figure 3). After Psa infection, tens of PPRs were differentially expressed in both ‘HongYang’ and ‘HuaTe’. A total of 45 differentially expressed PPRs were identified in both kiwifruits, with eight differentially expressed PPRs shared by both species. Additionally, 26 PPRs were specific to ‘HuaTe’, and 10 were specific to ‘HongYang’. The shared eight differentially expressed PPRs were *Actinidia05776*, *Actinidia08478*, *Actinidia18849*, *Actinidia19198*, *Actinidia24301*, *Actinidia25020*, *Actinidia28434*, and *Actinidia29423*, and they are labeled in Table S9. Out of the 45 differentially expressed PPRs, 18 were identified in ‘HongYang’, with 4, 10, 13, and 10 differentially expressed PPRs at 12 h after infection (hai), 24 hai, 48 hai, and 96 hai, respectively. ‘HuaTe’ exhibited more differentially expressed PPRs, with 34 identified (Table S9). In ‘HuaTe‘, there were 10, 14, 21, and 20 differentially expressed genes detected at 12 hai, 24 hai, 48 hai, and 96 hai, respectively. Furthermore, the fold changes of differentially expressed PPRs in ‘HuaTe’ were larger than those in ‘HongYang’. For both kiwifruits, the highest number of differentially expressed PPRs was observed at 48 hai after infection, indicating a critical immune response at this time point. In terms of the up–down-regulation trend of genes, more differentially down-regulated PPRs were found in both kiwifruits than differentially up-regulated PPRs, especially in ‘HuaTe’. In ‘HuaTe’, the number of differentially down-regulated PPRs was 5, 10, 14, and 12 at 12 hai, 24 hai, 48 hai, and 96 hai, respectively, while the up-regulated PPRs in ‘HuaTe’ totalled 5, 4, 7, and 8 at 12 hai, 24 hai, 48 hai, and 96 hai, respectively. In ‘HongYang’, the numbers of differentially down-regulated PPRs were 4, 6, 9, and 6 at 12 hai, 24 hai, 48 hai, and 96 hai, respectively, while the numbers of up-regulated PPRs in ‘HongYang’ were 0, 4, 4, 4 at 12 hai, 24 hai, 48 hai, and 96 hai, respectively. These observations indicate that down-regulated expression is the overall trend in response to Psa infection. We selected five differentially expressed PPRs as illustrations; they were all down-regulated in ‘HuaTe’ after Psa infection but expressed stably in ‘HongYang’ (Figure 3b). For example, *Actinidia18966.t1* and *Actinidia05278.t1* were differentially expressed at 24 hai, 48 hai, and 96 hai only in ‘HuaTe’. Thus, in comparison with ‘HongYang’, ‘HuaTe’ exhibited more differentially expressed PPRs, especially those that were down-regulated. Therefore, we speculate that the down-regulation of PPRs may be related to the resistance of ‘HuaTe’ to Psa.

Considering the differential expression levels of PPRs between the two kiwifruits under Psa infection, we further explored the corresponding chloroplast RNA editing events (Table S10), as shown in Figure 3c. A total of 61 RNA editing sites occurring in 29 genes were detected in this study. We observed a reduction in editing efficiency or a loss of editing in samples after Psa infection, especially in ‘HuaTe’. We selected six RNA editing sites with significantly reduced editing efficiency to illustrate this point: ndhB-277, rps2-83, matK-152, ndhD-293, petL-2, and rpoB-184. Editing at sites ndhD-293 and petL-2 was reduced at 12 hai, whereas editing at sites ndhB-277 and rps2-83 was completely lost at 12 hai. However, in ‘HongYang’, these notable reductions in editing were not detected. Additionally, the differentially expressed PPRs were mostly located in chloroplasts, which might thereby affect these chloroplasts’ RNA editing. Taken together, the above results showed that PPRs were responsive to Psa infection and exhibited significantly different expression levels between resistant and susceptible kiwifruits, suggesting the roles of PPRs in disease resistance. Compared with ‘HongYang’, the resistant kiwifruit ‘HuaTe’ exhibited a more dramatic response to Psa infection in not only in the gene expression of the PPRs but also RNA editing levels. Under pathogen stress, PPRs tend to be down-regulated, thereby reducing the RNA editing level to trigger downstream defense responses. This is consistent with our previous study about the roles of MORFs in kiwifruit [16].

### 2.4. Upstream Transcription Factors Associated with PPRs in Kiwifruit

**Figure 4:**
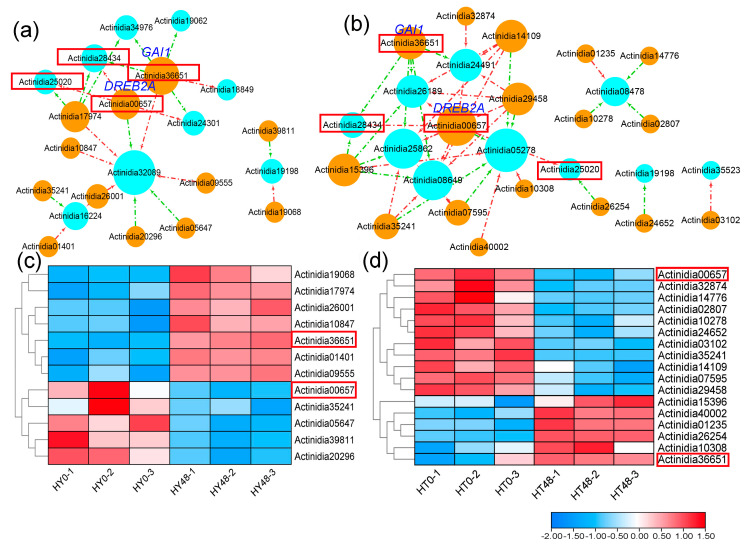
Upstream transcription factors associated with PPRs in kiwifruit. The regulatory networks between differentially expressed PPRs and transcription factors at 48 hai in ‘HongYang’ and ‘HuaTe’ are shown in (**a**) and (**b**), respectively. The nodes indicate PPRs and transcription factors, denoted by cyan-blue and orange circles, respectively. The shared upstream TFs and PPRs are highlighted with red boxes, and the gene names of TFs are labeled. The differential expression patterns of TFs at 48 hai in ‘HongYang’ and ‘HuaTe’ are shown in (**c**) and (**d**), respectively. The shared upstream TFs and PPRs are highlighted with red boxes.

To explore the underlying pathways that may regulate the expression of PPRs in kiwifruit, we selected the differentially expressed PPRs at 48 hai and obtained their upstream transcription factors (TFs) from the PlantRegMap database, only keeping those that demonstrated differential expression at 48 hai. Finally, we constructed the regulatory network of PPRs and upstream TFs, consisting of 9 differentially expressed PPRs and 12 upstream TFs in ‘HongYang’, and 10 differentially expressed PPRs and 17 upstream TFs in ‘HuaTe’, as shown in Figure 4. These upstream TFs were up-regulated or down-regulated at 48 hai and positively or negatively regulated the gene expression of PPRs in ‘HongYang’ and ‘HuaTe’. There were two shared upstream TFs (*Actinidia00657* and *Actinidia36651*) that regulated the expression of differentially expressed PPRs (*Actinidia25020* and *Actinidia28434*) in both ‘HongYang’ and ‘HuaTe’, indicating their common regulatory function. One TF gene, *Actinidia00657*, encoding dehydration-responsive element-binding protein 2C (DREB2A), was down-regulated in both ‘HongYang’ and ‘HuaTe’ and negatively regulated the expression of the PPR gene *Actinidia25020* at 48 hai. Another upstream TF gene, *Actinidia36651*, encoding the DELLA protein (GAI1), which acts as a repressor of the gibberellin (GA) signaling pathway, was up-regulated in both ‘HongYang’ and ‘HuaTe’ and positively regulated the expression of the PPR gene *Actinidia28434*. These differentially expressed TFs may regulate the expression of PPR genes, thereby affecting RNA editing under pathogen stress.

## 3. Discussion

Due to its richness in vitamins and antioxidants, kiwifruit has become a popular horticultural fruit with high economic value. However, various diseases threaten its yield and quality. In plants, RNA editing acts as a post-transcriptional modification and plays multiple roles in the processes of growth, development, and stress response. PPRs are important subunits of RNA editing machinery and some function as organelle-specific RNA-binding proteins, thereby playing a crucial role in RNA editing regulation. PPRs are extensively distributed across plant lineages, comprising more than 400 family members [41]. Some PPRs in Arabidopsis, such as SOAR1 [42] and PPR96 [43], are involved in the response to stress. However, the functions of PPRs and their response to stress in kiwifruit have rarely been reported. In this study, we investigated the structure, classification, and expression of the PPR family in two representative kiwifruit species (*Ach* and *Ace*) with different disease resistance. A total of 497 and 499 PPRs were identified in ‘HongYang’ and ‘HuaTe’, respectively. The results indicated that the expansion of the kiwifruit PPR family mainly resulted from segmental duplication. The comparable number of families in the two kiwifruits suggest that the expansion of the PPR gene family occurred before species differentiation. Most kiwifruit PPRs were predicted to be localized in mitochondria or chloroplasts, which is consistent with studies of PPRs in other plants.

Based on RNA-Seq data during kiwifruit’s fruit development and maturation, we observed differential expression patterns of PPRs at various time points, suggesting their involvement in fruit development. Additionally, we conducted further transcriptome analysis using RNA-seq data from resistant (‘HuaTe’, *Ace*) and susceptible (‘HongYang’, *Ach*) kiwifruits during the early stages of Psa infection. This analysis allowed us to investigate the expression of PPRs between these two different kiwifruit varieties. For both kiwifruit varieties, the largest number of differentially expressed PPRs was observed at 48 hai after infection, indicating a critical immune response at this time point. These results indicated that PPRs also play a role in responding to Psa infection, highlighting their involvement in plant immunity. When comparing differentially expressed PPR genes, we found that more differentially down-regulated PPRs were detected than up-regulated ones in both kiwifruit varieties, particularly in ‘HuaTe’. Moreover, ‘HuaTe’ exhibited a greater number of differentially expressed PPRs compared to ‘HongYang’, with larger fold changes in gene expression. We also investigated the corresponding RNA editing events in chloroplasts and observed a reduction in or loss of editing efficiency in some sites, particularly in ‘HuaTe’, following Psa infection. Based on these results, we propose that PPRs may play a role in regulating RNA editing and the response to disease resistance in kiwifruit. Furthermore, we identified several transcription factors associated with PPRs, including *DREB2A*, and *GAI1*. These differentially expressed transcription factors may regulate the expression of kiwifruit PPRs in response to both development processes and stress conditions.

In our previous studies [16], we observed differential expression of *MORF* genes in ‘HuaTe’ and ‘HongYang’ following pathogen infection. Specifically, *MORF2.1*, *MORF9.1*, and *MORF7* in ‘HuaTe’ were significantly down-regulated, which is consistent with the expression patterns of PPRs analyzed in this study. Furthermore, we conducted an investigation into the upstream transcription factors (TFs) of both *PPR* and *MORF* genes in kiwifruit (Figure 3b and Figure S3). This regulatory network included 49 edges and 24 nodes, with nine TFs displaying differential expression. Notably, six out of these TFs were significantly up-regulated. One of the co-regulating TFs, *Actinidia17974.t1*, encodes *Transcription factor IIIA*, which is known for its role in regulating 5S rRNA levels during development. Other TFs identified belong to the C2H2 zinc finger gene family or BCR-BPC gene family, with functions related to transcription factor activity, sequence-specific DNA binding, 5SrRNA binding, developmental process, and metal ion binding. Interestingly, the majority of the differentially expressed TFs were exclusive to ‘HuaTe’, including *Actinidia10847.t1*, *Actinidia26001.t1*, *Actinidia17974.t1*, and *Actinidia39948.t1*. These four transcription factors specifically responded to pathogen infection in ‘HuaTe’ but not in ‘HongYang’. It is plausible to speculate that these transcription factors may play significant roles in pathogen resistance in ‘HuaTe’. Both *PPR* and *MORF* genes exhibited a tendency to be down-regulated in response to Psa infection, leading to the loss of and reduction in RNA editing, especially in ‘HuaTe’, which demonstrated higher resistance to Psa. These findings collectively suggest that RNA editing factors play crucial roles in plant immunity. In comparison to ‘HongYang’, resistant kiwifruit displayed a more pronounced response to Psa infection, both in terms of RNA editing level and gene expression. Therefore, we hypothesize that *PPRs* and *MORF* genes work together during the pathogen infection process. Under pathogen-induced stress, similar to *MORF* genes, PPRs tend to be down-regulated, resulting in reduced RNA editing levels and the initiation of downstream defense responses. In summary, our results provide insights into the evolution of PPRs and their involvement in pathogen stress responses in kiwifruit. However, it is important to note that our study primarily relies on genomic and transcriptomic data analysis using computational methods. There are limitations due to the lack of experimental validation. Further direct evidence through mutants or knockdown experiments is necessary to establish a causal link between PPRs and phenotypes.

**Figure 5:**
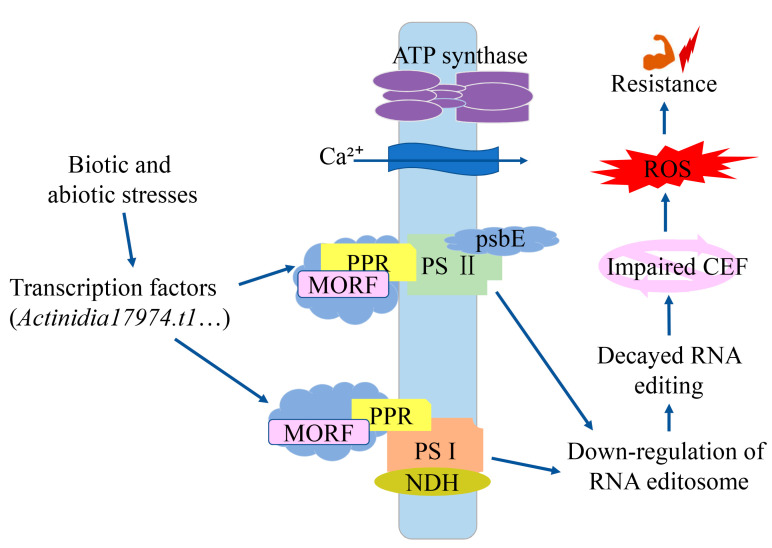
Schematic model for the roles of PPRs in kiwifruit. Under pathogen stress, the expression of PPRs and other editing factors, such as MORF genes, tends to be down-regulated. This down-regulation leads to a reduction in RNA editing, impaired CEF, and an increased level of ROS, ultimately enhancing resistance to pathogens. CEF: cyclic electron flow; PS I: photosystem I complex; PS II: photosystem II complex.

Being intra-cellular energy delivery sites, mitochondria and chloroplasts are important sources of reactive oxygen species (ROS), which act as key defense molecules in the plant immune response [44]. Therefore, mitochondria and chloroplasts play key roles in the interactions between pathogens and hosts. However, how proteins localized in mitochondria and chloroplasts regulate the plant immune system remains unclear. There is increasing evidence to suggest that many PPRs are involved in responses to multiple stresses. For instance, PPR40 in Arabidopsis has been shown to provide signaling connections between mitochondrial electron transport elements, and the knockout of the *PPR40* gene leads to the accumulation of ROS, increased lipid peroxidation, and superoxide dismutase activities [45]. In this study, the comparison of PPRs and RNA editing between resistant and susceptible kiwifruit also confirmed the roles of mitochondria and chloroplasts in disease resistance. Under pathogen infection, we observed the down-regulated expression of *PPRs* and *MORF* genes (*MORF9.1*, *MORF7*, and *MORF2.1*), as well as decreased RNA editing in resistant kiwifruit. *ndhB* encodes the subunit B of the chloroplast NADH dehydrogenase-like complex (NDH), which constitutes an important component of cyclic electron flow (CEF) in photosystem I. Therefore, we speculated that the reduced editing efficiency of these genes may trigger impaired CEF, leading to the activation of reactive oxygen-mediated retrograde signaling and significantly enhanced disease resistance to pathogens (Figure 5). *PPR* and *MORF* genes may regulate plant-pathogen interactions by controlling the degree of RNA editing, particularly the composition of the NDH complex. However, further rigorous experimental validation is needed to substantiate the physiological relevance and this proposed model.

## 4. Methods and Materials

### 4.1. Genome-Wide Identification of PPRs in Ach and Ace

The genome sequence and annotation files of *Ach* and *Ace* were retrieved from NCBI (https://www.ncbi.nlm.nih.gov/, accessed on 1 January 2022), with versions ‘ASM966300v1’ and ‘White_v1.0’, respectively. Using the known 450 PPRs in Arabidopsis as the query reference [46], including PP438_ARATH, PP264_ARATH, etc., of the P-type, and PP320_ARATH and PP207_ARATH, etc., of the PLS-type, we employed two search strategies to obtain kiwifruit PPRs. First, we conducted a BLASTP v2.13.0+ search against the complete genome with an E-value of 0.00001. Second, we constructed the Hidden Markov Model (HMM) profiles of PPRs in Arabidopsis to search against the two kiwifruit protein databases using HMMER v3.4 software with an E-value 0.001 [34]. Finally, we verified all hits by checking the existence of PFAM (http://pfam.sanger.ac.uk/, accessed on 15 January 2022) domain PF01535, and sequences containing fewer than two PPR motifs were excluded. The phylogenetic tree was constructed using the maximum likelihood (ML) method implemented in MEGA v5.2 [38]. Based on the phylogenetic relationships of known representative PPRs in Arabidopsis from different clades, all the PPRs were classified into different types.

### 4.2. Subcellular Localization and Physical Localization

We used TargetP v2.0 [35], Predotar v1.3 [36], and PSORT (https://www.genscript.com/psort.html, accessed on 1 May 2022) to predict the putative subcellular localization of kiwifruit PPRs. In addition, the physical distribution of the PPRs on chromosomes was obtained from the genome annotation files, and a sketch map of the genes’ physical locations was drawn using TBtools v2.001 [47].

### 4.3. Synteny Analysis of PPRs in Two Kiwifruit Species

We conducted a self-blast by comparing proteins against their respective genomes using BLASTP v2.13.0+ with an E-value of 0.00001. All BLASTP hits were then used as inputs for the MCScanX v1.0 [37] software to identify possible collinear blocks within the genomes of *Ach* and *Ace*. Based on the self-blast results, we used the ‘Duplicate_gene_classifier’ command of MCScanX to clarify the origins of the duplicate genes into several types, including whole-genome/segmental, tandem, proximal, or dispersed duplications. All intragenomic synteny relationships were visualized using Tbtools v2.001 [47].

### 4.4. Expression Analysis of PPR Genes in Ach and Ace

To examine the expression profiles of kiwifruit PPRs, we performed transcriptome analysis using two sets of RNAseq data. For *Ach*, RNA-seq data of fruit samples were retrieved from the NGDC database (https://ngdc.cncb.ac.cn/, accessed on 1 January 2022) under the accession number PRJCA003268 [39]. Six developmental periods (40, 60, 80, 100, 120, and 140 days after pollination) and six maturity periods (4, 6, 8, 10, 12, and 14 days after harvest of ripe fruit) were selected for ‘HongYang’. Each time point consisted of three replicates. For both *Ach* and *Ace*, RNA-seq data of leaves in response to Psa after early infection were retrieved with accession number PRJNA514180 [40]. Transcriptome analysis was performed using the methods described in a previous study [16]. We measured gene expression levels in fragments per kilobase of transcript per million mapped reads (FPKM) and identified the differentially expressed genes using EdgeR (https://bioconductor.org/packages/release/bioc/html/edgeR.html, accessed on 1 July 2022). A heatmap of gene expression was generated using Tbtools v2.001 [47].

### 4.5. Gene Co-Expression Network Analysis

Using RNA-seq data obtained from fruit samples of *Ach*, we employed WGCNA v1.0 [48] to identify co-expression modules and key PPRs associated with fruit development and maturation. We set the soft threshold β to 16. Initially, we converted the adjacency matrix of PPR expression into a topological overlap matrix (TOM). Subsequently, we calculated the characteristic genes and performed hierarchical clustering (with a mergeCutHeight value of 0.25) to identify key modules. Module signature genes (ME) and module members (MM) were employed to distinguish important modules associated with fruit development and maturation. ME represents the primary component within a module and characterizes the module’s representation pattern. MM represents the relationships between genes and module-characteristic genes and assesses the reliability of genes as part of the module.

### 4.6. Identification of RNA Editing Sites

To begin, we mapped all the RNA-seq data against the chloroplast genome reference using HISAT2 v2.2.1 software with default parameters [49]. We utilized ‘bcftools’ tool to detect variants/SNPs and generate VCF files [50]. Consequently, RNA editing sites were filtered based on the variant results. Using the SNP-calling results and genome annotation files, we identified RNA editing sites and their corresponding information using the REDO tool v1.0 [51]. As a comprehensive application tool, REDO can accurately identify plant RNA editing sites from RNA-seq data. We applied a series of comprehensive rule-dependent and statistical filters provided by the REDO tool to reduce false positives. Additionally, we manually examined all mismatches to minimize false-positive sites. RNA editing efficiency was defined as the proportion of edited transcripts among the total covered transcripts.

### 4.7. Identification of Upstream Regulatory Transcription Factors

We employed the PlantRegMap database (http://plantregmap.gao-lab.org/, accessed on 10 July 2022) to retrieve the transcriptional regulatory map of kiwifruit [52]. The database contains previously identified transcriptional regulations for numerous plant species, inferred from the literature, ChIP-seq data, and predictions from transcription factor (TF) binding motifs and regulatory element data. Subsequently, we annotated the regulatory transcription factors through blast results against reference proteins in Uniprot (https://www.uniprot.org/, accessed on 20 July 2022), and we visualized the regulatory relationships using Cytoscape v3.8.2 [53].

## 5. Conclusions

In conclusion, this study analyzed the chromosomal positions, phylogenetic relationships, and evolution of PPRs in *Ach* and *Ace*, and provided their expression patterns at different stages of fruit development and under pathogen stress. The results showed that PPRs were differentially expressed at various stages of fruit development and maturation, indicating their role in kiwifruit’s fruit development and maturation. Differences in the expression and RNA editing profiles of PPRs between resistant and susceptible kiwifruit were observed after pathogen infection, indicating the roles of PPRs in the stress response. Similar to *MORF* genes, PPRs were also associated with resistance and influenced RNA editing sites in chloroplasts. This suggests that RNA editing involving *PPR* and *MORF* genes may be linked to chloroplast-mediated immunity. The findings of this study will serve as a reference for further understanding the molecular mechanism of plant immunity and for breeding kiwifruit varieties with enhanced resistance.

## Figures and Tables

**Table 1:** Distribution of different types of PPRs in two kiwifruit species.

Type	*Actinidia chinensis* (*Ach*)		*Actinidia eriantha* (*Ace*)	
	Number	Percentage	Number	Percentage
P	251	50.5%	247	49.50%
E	91	18.31%	74	14.83%
E+	95	19.11%	102	20.44%
DYW	60	12.07%	76	15.23%
All	497	/	499	/

## Data Availability

All raw reads used in this work were deposited in NCBI Bio-Project under the accession numbers PRJCA003268 and PRJNA514180.

## Supplementary Materials

The supporting information can be downloaded at: https://www.mdpi.com/article/10.3390/ijms241813700/s1.

## Abbreviations

PPRs: pentatrico peptide repeat proteins; MORF: multiple organelle RNA editing factors; Psa: *Pseudomonas syringae* pv. *Actinidiae*; C-to-U: cytidines substituting uridines; RNA-seq: RNA sequencing; SNPs: single-nucleotide polymorphisms; WGS: whole-genome re-sequencing; cox1: cytochrome oxidase subunit 1; nad: NADH dehydrogenase; rps: ribosomal protein gene; *rps14*: *30S ribosomal gene*; *ccmB*: *cytochrome c maturation gene*; hai: hours after inoculation; HMM: Hidden Markov Model; CEF: cyclic electron flow; PS I: photosystem I complex; PS II: photosystem II complex; *Ach*: *Actinidia chinensis*; *Ace*: *Actinidia eriantha*; TFs: transcript factors.
