# Supplementary material for: A Genome-Wide Analysis of the Pentatricopeptide Repeat Protein Gene Family in Two Kiwifruit Species with an Emphasis on the Role of RNA Editing in Pathogen Stress
Source: Int J Mol Sci. 2023 Sep 5;24(18):13700. doi: 10.3390/ijms241813700 (PMC10530749; doi:10.3390/ijms241813700)
Supplement: Supplementary file 1 [file ijms-24-13700-s001.zip › SupplementaryFigures.pdf]

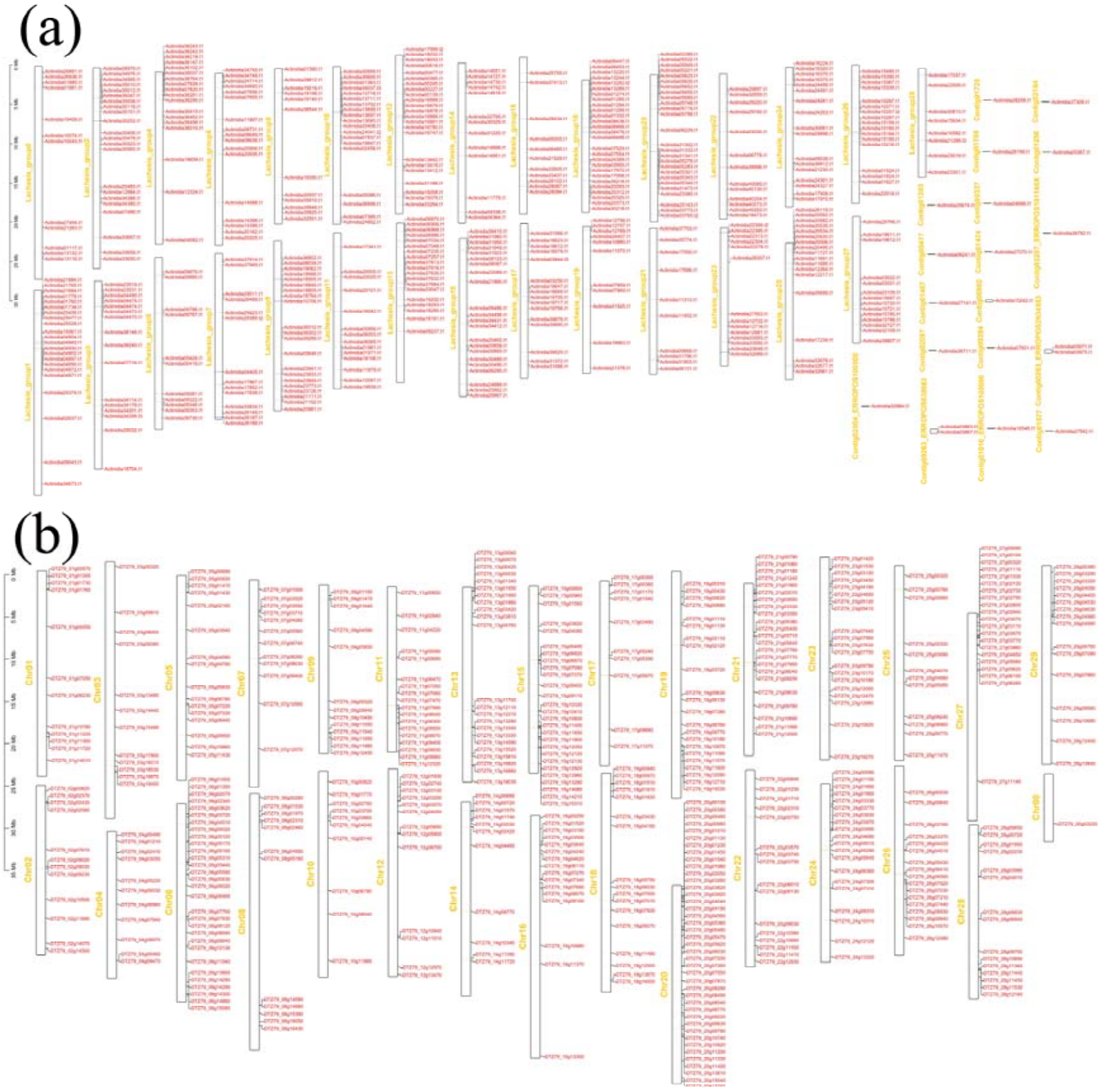

Figure S1. Chromosomal locations of *PPR* genes in kiwifruit. The chromosomal locations of the *PPR* genes were mapped with TBtools v2.001 [1]. (a) Chromosomal locations of *PPR* genes in *Actinidia chinensis*. (b) Chromosomal locations of PPR genes in *Actinidia eriantha.*

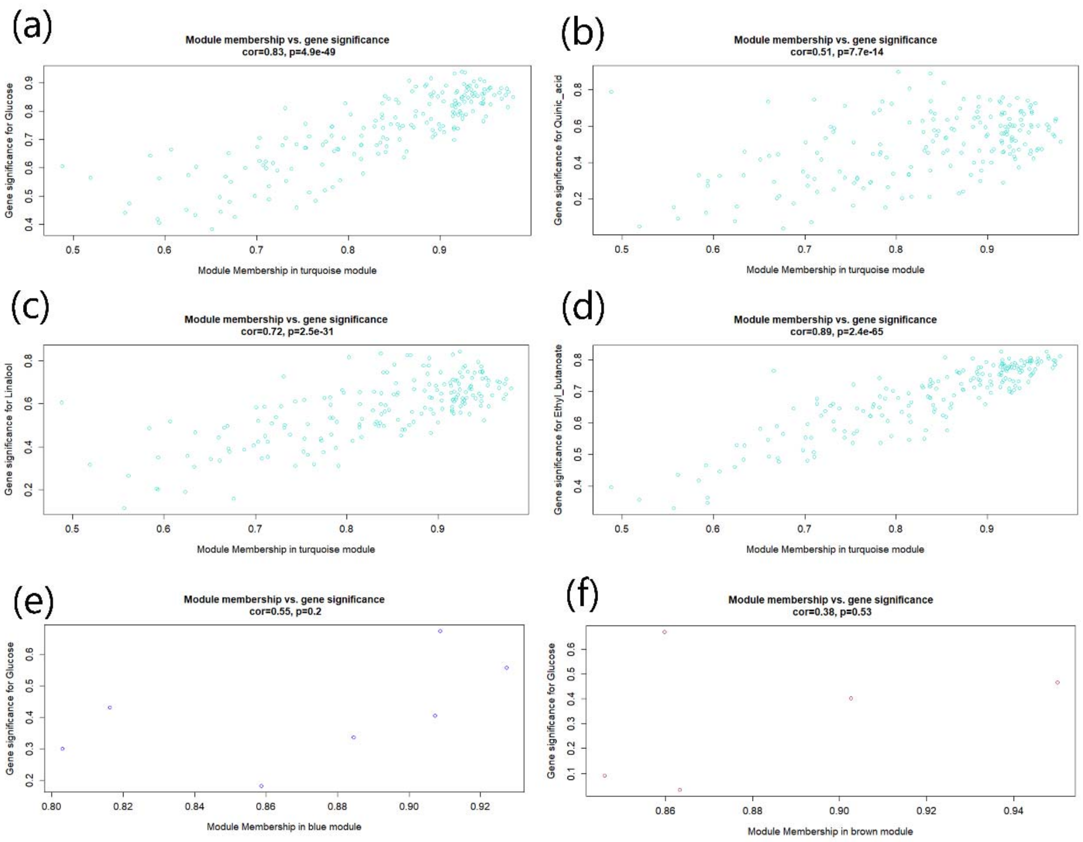

Figure S2. Module membership-gene characteristics associated with glucose, quinic acid, linalool, and ethyl butyrate in kiwifruit.

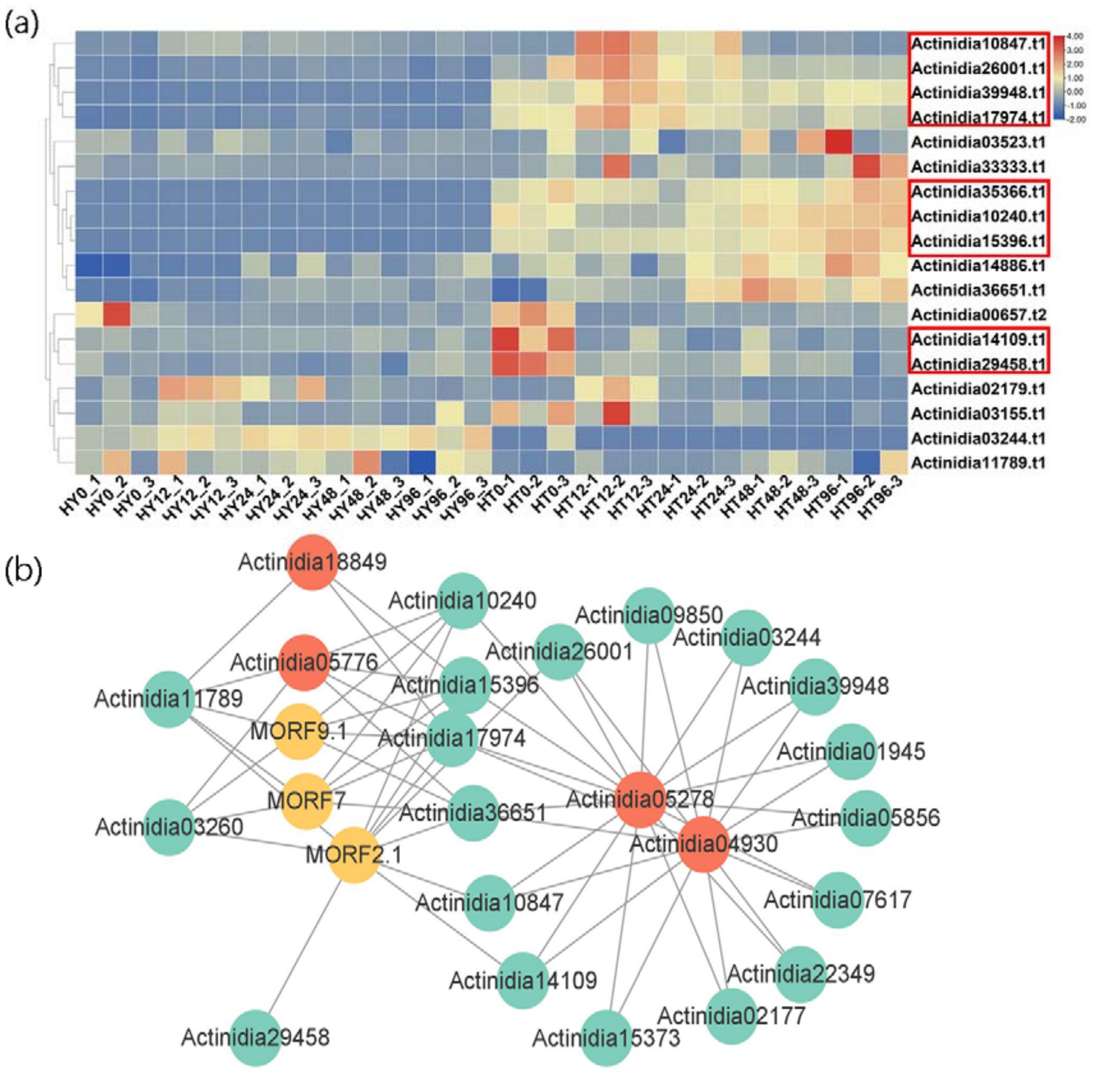

Figure S3. Expression and interaction network of upstream transcription factors that regulated kiwifruit *PPR* genes and the *MORF* genes. (a) Gene expression profiles of the upstream transcription factors of kiwifruit *PPR* genes and the *MORF* genes. Transcription factors that are differentially expressed are indicated in red boxes. (b) The regulation network of upstream transcription factors with *PPR* genes and *MORF* genes. *PPR*, *MORF* and transcription factors genes are shown in orange, yellow, and turquoise, respectively.

Reference

1. Chen C, Chen H, Zhang Y, Thomas HR, Frank MH, He Y, Xia R: **TBtools: An Integrative Toolkit Developed for Interactive Analyses of Big Biological Data**. *Molecular plant* **2020**, *13*, 1194–1202.
